# Challenges to the Application of Spatially Explicit Stochastic Simulation Models for Foot-and-Mouth Disease Control in Endemic Settings: A Systematic Review

**DOI:** 10.1155/2020/7841941

**Published:** 2020-11-21

**Authors:** Muhammad Usman Zaheer, Mo D. Salman, Kay K. Steneroden, Sheryl L. Magzamen, Stephen E. Weber, Shaun Case, Sangeeta Rao

**Affiliations:** ^1^Animal Population Health Institute, Department of Clinical Sciences, College of Veterinary Medicine and Biomedical Sciences, Colorado State University, Fort Collins CO 80523, USA; ^2^FMD Project Office, Food and Agriculture Organization of the United Nations, ASI Premises, NARC Gate # 2, Park Road, Islamabad 44000, Pakistan; ^3^Department of Environmental and Radiological Health Sciences, College of Veterinary Medicine and Biomedical Sciences, Colorado State University, Fort Collins CO 80523, USA; ^4^Department of Civil and Environmental Engineering, Walter Scott, Jr. College of Engineering, Colorado State University, Fort Collins CO 80521, USA

## Abstract

Simulation modeling has become common for estimating the spread of highly contagious animal diseases. Several models have been developed to mimic the spread of foot-and-mouth disease (FMD) in specific regions or countries, conduct risk assessment, analyze outbreaks using historical data or hypothetical scenarios, assist in policy decisions during epidemics, formulate preparedness plans, and evaluate economic impacts. Majority of the available FMD simulation models were designed for and applied in disease-free countries, while there has been limited use of such models in FMD endemic countries. This paper's objective was to report the findings from a study conducted to review the existing published original research literature on spatially explicit stochastic simulation (SESS) models of FMD spread, focusing on assessing these models for their potential use in endemic settings. The goal was to identify the specific components of endemic FMD needed to adapt these SESS models for their potential application in FMD endemic settings. This systematic review followed the PRISMA guidelines, and three databases were searched, which resulted in 1176 citations. Eighty citations finally met the inclusion criteria and were included in the qualitative synthesis, identifying nine unique SESS models. These SESS models were assessed for their potential application in endemic settings. The assessed SESS models can be adapted for use in FMD endemic countries by modifying the underlying code to include multiple cocirculating serotypes, routine prophylactic vaccination (RPV), and livestock population dynamics to more realistically mimic the endemic characteristics of FMD. The application of SESS models in endemic settings will help evaluate strategies for FMD control, which will improve livestock health, provide economic gains for producers, help alleviate poverty and hunger, and will complement efforts to achieve the Sustainable Development Goals.

## 1. Introduction

Foot-and-mouth disease (FMD) is endemic in different parts of the world [1–11] and is associated with substantial economic losses [12–14]. The costs associated with production losses and vaccination in endemic regions amount to USD 6.5-21 billion, and the annual outbreak-associated losses in FMD-free countries and zones exceed USD 1.5 billion [14]. International organizations such as the Food and Agriculture Organization of the United Nations (FAO), the World Organization for Animal Health (OIE), and the European Commission for the Control of Foot-and-Mouth Disease (EuFMD) have called for a more targeted control strategy in the “Progressive Control Pathway for FMD” (PCP-FMD) to reduce the disease burden and minimize economic costs associated with it [15–19].

Simulation modeling has become common to investigate the spread of highly contagious diseases, assist in policy-making, and act as a decision support tool [20–24]. These models can be categorized as deterministic or stochastic based on how they incorporate variability and uncertainty and nonspatial or spatially explicit based on how they treat spatial relationships [25]. Spatial models require “locational data” for herds of animals and incorporate spatial proximity and spatial relationships in estimating disease risk [25]. The spatially explicit stochastic simulation (SESS) models incorporate uncertainty in the input and output parameters, heterogeneity in disease processes, and integrate geographic locations and spatial proximity of herds that affect their relative exposure and transmission risk [25, 26]. Considering the epidemiology and ecology of FMD, these models are most appropriate for simulating FMD spread in endemic and free settings.

Many models have been developed to mimic the spread of FMD in specific regions or countries [27–31]. These models have been used to conduct risk assessments, analyze outbreaks using historical data or hypothetical scenarios, assist in policy decisions during outbreaks, policy formulation in preparedness planning, and evaluate economic impacts [32–39]. In disease-free countries, models are used to identify gaps in preparedness, such as estimating required resources [40, 41]. In endemic countries, models can be useful for comparing mitigation strategies to guide future FMD control [42].

However, most of the reported literature on FMD simulation models is associated with disease-free countries with minimal application of these models in countries with an endemic status of FMD [43]. The development of simulation models of infectious livestock diseases such as FMD in endemic settings is enormously challenging for reasons such as lack of interest and understanding of perceived needs, political or economic constraints, insufficient data to support model parameters, and complex epidemiology of FMD in endemic settings [44, 45]. For instance, routine prophylactic vaccination (RPV) is practiced in endemic settings to control FMD, but factors such as the duration of natural immunity, the rate of vaccine-induced antibody waning, and the rate of disease reintroduction influence FMD control and are critical determinants of the success of vaccination programs [46]. Also, the circulation of multiple FMD virus serotypes is a common characteristic in endemic settings [47, 48]. The transmission patterns and duration of immunity are variable for different serotypes [49].

Given the epidemiology of FMD, a SESS model for FMD endemic settings should have the ability to model FMD progression for multiple cocirculating serotypes, a range of control options such as emergency vaccination, RPV, stamping-out, and ability to incorporate population dynamics during the simulations [46–49]. Application of simulation models to endemic settings would be beneficial in advancing our knowledge, understanding FMD dynamics, and facilitating both local and global control of FMD [43].

This paper's objective was to report the findings from a study conducted to review the existing published original research literature on SESS models of FMD spread, focusing on assessing these models for their potential use in endemic settings. The goal was to identify the specific components of endemic FMD needed to adapt the SESS models for use in FMD endemic settings, which will help evaluate strategies for FMD control, improve livestock health, provide economic gains for producers, help alleviate poverty and hunger, and will complement efforts to achieve the Sustainable Development Goals.

## 2. Materials and Methods

### 2.1. Protocol

This systematic review follows the guidelines established in the Preferred Reporting Items for Systematic Reviews and Meta-Analyses (PRISMA) [50].

### 2.2. Definition of SESS

For this systematic review, the definition of a SESS model was developed to facilitate the process of identifying the search items that could be included in the qualitative synthesis [25, 26]. A SESS model was defined as “one that takes input parameters in the form of statistical distributions, consequently generating a distribution of values for results, and incorporates geographic locations and spatial proximity of animals or herds that affect their relative exposure and transmission risk”.

### 2.3. Eligibility Criteria

Any original research in the English language published during any timespan that described or used a SESS model for simulating FMD spread or evaluating mitigation in any part of the world would be included.

### 2.4. Information Sources and Search Strategy

#### 2.4.1. Databases

Three databases, i.e., Google Scholar (GS), PubMed, and Web of Science (WoS), were chosen to identify the relevant literature.

#### 2.4.2. Search Strategy

All three databases were searched by the primary author on the same day, i.e., October 7, 2018, to identify the literature. The keywords used were *((foot and mouth disease OR FMD OR FMDV) AND (stochastic AND simulation))*. These keywords were consistent across all databases searched. These keywords were chosen to be sensitive in capturing all possible publications containing these keywords. The resulting citations were managed in Microsoft Excel (2016).

### 2.5. Screening and Inclusion/Exclusion Criteria

**Figure 1:**
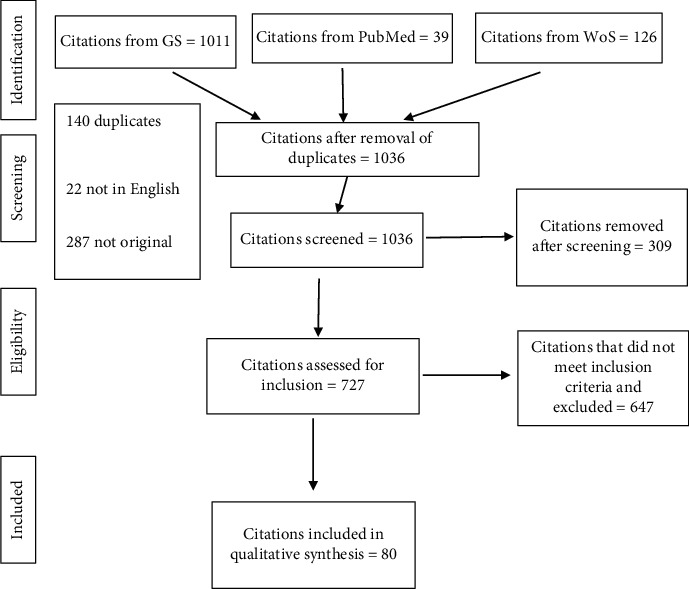
Flow diagram of the literature search, screening, and inclusion/exclusion criteria (adapted from [50]).

The citations identified through the database search were first screened (steps 1–3) and then assessed to identify SESS models for FMD (step 4). Eventually, a flowchart was created (Figure 1) as per PRISMA guidelines [50].

#### 2.5.1. Screening Criteria

The search results were screened in three steps. In step 1, citations were checked for duplicates across databases and within the database. All duplicates were removed from the pool of citations at this stage. In the second step, citations were screened for their publication language. Citations not in the English language were removed. In step 3, citations were checked for their document type. Only the original research publications were retained, and all other document types were excluded at screening step because of the inability to evaluate the model's features and their application.

#### 2.5.2. Inclusion Criteria

In step 4, the title and abstract of the remaining citations were evaluated to determine if they contained the word(s) foot-and-mouth disease, FMD, or FMDV. If citations included these keywords, they were evaluated to determine if they describe or use a SESS model to understand FMD spread and evaluate mitigation strategies. Citations that did not meet this criterion were excluded, and the remaining citations were selected for qualitative synthesis. The rationale for this strict criterion was to move from being sensitive as indicated above to be specific to the disease of interest, i.e., FMD.

The remaining citations were grouped based on the unique SESS model they described or used. An additional group was created for citations that compared different models to evaluate alternative mitigation strategies and aid in decision-making.

### 2.6. Assessment of Final Citations and Data Extraction

Each unique SESS was then evaluated for its assumptions and epidemiologic design, emphasizing its application in endemic settings. Specifically, each SESS model was assessed for its ability to model multiple FMD virus serotypes in parallel, the range of mitigation strategies (emergency vaccination, RPV, and stamping-out), and livestock population dynamics. These factors were chosen because of their epidemiologic significance in endemic FMD.

After evaluating each SESS, the data on various features were extracted and tabulated to compare different SESS. Each SESS was then summarized, and limitations in each SESS were highlighted. Finally, suggestions were made for adaptation of the SESS models for their potential use in endemic settings.

## 3. Results

Figure 1 shows the flow chart that summarizes the process of identifying citations from different databases, screening of citations, and assessment against inclusion criteria.

### 3.1. Database Searches and Screening

The database search resulted in 1176 accessible citations: 1011, 39, and 126 from GS, PubMed, and WoS, respectively. In step 1, all 39 citations from PubMed and 97 citations from WoS were identified as duplicate with GS citations. Out of 1011 GS citations, four were duplicated within GS. All 140 duplicate citations were removed.

In step 2, the remaining 1036 citations were screened for their publication language, and 22 were removed because they were not in the English language. In step 3, the remaining 1014 citations were checked for their document type, and 287 were excluded because these were not published original research.

### 3.2. Inclusion Criteria

After screening of the remaining 727 citations, 647 were excluded because these citations either did not contain the word(s) foot-and-mouth disease, FMD, and FMDV in their title or abstract or did not describe or use a SESS for FMD and hence failed to meet the inclusion criteria. The remaining 80 citations were included in the qualitative synthesis. Excluded models were either not stochastic or did not include a spatial component.

### 3.3. Unique SESS Models

Nine unique SESS models were identified, and relevant citations were grouped in Table 1. Each unique SESS model was assessed for its assumptions and epidemiologic design with specific emphasis on its application in endemic settings.

### 3.4. Top Five SESS Models

Below is a short description of the top five SESS models (based on the number of citations identified in the study), emphasizing their suitability for the general aim of this study. For a more detailed description of these SESS models, readers are referred to the original citations specified in Table 1.

#### 3.4.1. Warwick Model

In response to an outbreak of FMD that hit the UK in 2001, a stochastic spatial model was developed to simulate between farm spread of FMD [35]. The model was designed to act as a decision support tool during the 2001 epidemic. Since then, this model has undergone various adaptations [51, 52] and is now termed the Warwick model.

The Warwick model has been used to understand predictors of FMD transmission risk [53], identify high-risk areas [54], understand spatiotemporal process [55], evaluate mitigation strategies [56, 57], determine optimal control strategies [58, 59], guide policymakers [60], assist in real-time policy-making [61], understand the effect of vaccine availability constraints on epidemiologic and economic outcomes [62], estimate prevalence of asymptomatic carriers [63], understand the effect of livestock density vs. farm density [64], assess agreement between model outputs and epidemic data [65], understand the impact of the resolution of spatial data to inform control policies [66], and determine the predictor of final epidemic size [67] and computational advancement [68].

#### 3.4.2. DADS Model

The Davis Animal Disease Simulation (DADS) model is a stochastic, spatial simulation model to simulate the spread and evaluate the alternative mitigation strategies for FMD control in a designated geographical area [27, 32]. It has been used to estimate FMD spread [69, 70], examine epidemic and economic impacts [71], evaluate mitigation strategies [33, 72, 73], evaluate the effect of animal movement tracing [74], and examine the importance of stochasticity and modifying the assumption of homogeneous mixing [25].

An optimal control model was formulated based on the DADS structure to evaluate the control strategies for FMD in the USA [75, 76]. The DADS has been modified at the Technical University of Denmark, and the modified version is known as DTU-DADS. DTU-DADS is being used in FMD-free countries to understand the hypothetical spread of FMD, evaluate mitigation strategies, and help with contingency planning [77–80, 108].

#### 3.4.3. AusSpread Model

AusSpread is a stochastic, spatial simulation model that operates in a GIS environment to simulate the spread of FMD between herds [28, 81]. AusSpread is the outcome of more than ten years of extensive work of the Australian Government's Department of Agriculture, Fisheries, and Forestry [116, 117]. The intention behind this extensive effort was to have a model that could be used as a decision support tool for infectious diseases like FMD that pose the most significant economic threat to Australia [118].

Since the development of the AusSpread model, it has continuously been used in FMD-free regions to evaluate alternative mitigation strategies [82–84], assist in preparedness planning [85], estimate resources [40, 41], and evaluate the benefits of effective traceability system [86] and early detection [87].

#### 3.4.4. ISP Model

InterSpread Plus (ISP) is a stochastic, spatial simulation model of the between-farm spread of infectious diseases such as FMD [30]. The ISP model was developed to mimic the spread of FMD in New Zealand, a country free of FMD, to aid in preparedness planning and decision-making [30, 88]. The ISP model is a revised version of the InterSpread (IS) model that has been used to model alternative mitigation strategies during the 2001 FMD epidemic in the UK [37].

The ISP model has been used for FMD to evaluate alternative mitigation strategies [39, 89–91], assist in developing contingency plans [92], and evaluate the benefits of an effective traceability system [93].

#### 3.4.5. NAADSM Model

The North American Animal Disease Spread Model (NAADSM) is a stochastic, spatial model developed in the US to model the between-farm spread of infectious animal diseases such as FMD and CSF [29, 94].

The NAADSM is the only open-source SESS model with a user-friendly interface. It has been used in FMD-free settings to understand FMD spread and evaluate alternative mitigation strategies [95], identify optimal vaccination strategy [36], evaluate economic impacts [96, 97], and understand the effect of model complexity on model predictions [98].

### 3.5. Multiple SESS Models

As indicated in Table 1, ten citations reported using more than one SESS model. These studies ranged from model comparisons and country comparisons [80, 108–113, 119] to ensemble modeling and structured decision-making [114, 115]. The model comparison highlights the consistency in outcomes from commonly used SESS models. These findings are essential for increasing end-user confidence in model outcomes and their use in informed decision-making.

### 3.6. Assessment of SESS Models

The SESS models (Table 1) assessed in this systematic review are equipped with two important control options, i.e., emergency vaccination and stamping-out (Table 2), to simulate the impact of these strategies in epidemic settings, which are otherwise disease-free. “Routine prophylactic vaccination (RPV)” is practiced cyclically in endemic settings to control FMD, but the models lack this feature. Hence, application in endemic settings would require modification of SESS models to equip models to evaluate RPV's impact on FMD dynamics.

All the assessed SESS models were designed for and applied in FMD-free countries to simulate the spread of FMD and evaluate alternative mitigation strategies in the face of an incursion. In such an application, modelers assume that there is only one circulating serotype and uses the serotype's progression parameters. In endemic settings, however, cocirculation of multiple serotypes is a crucial component of FMD epidemiology, which needs to be added as an option for the modeler to include progression parameters for multiple serotypes in parallel (Table 2).

Additionally, the assessed SESS models (Table 2) do not consider population dynamics, i.e., births and deaths that might be occurring during the simulation, except in the case of stamping-out. Any application of these models in endemic settings would require the addition of population dynamic parameters.

Based on the assessment of SESS models through this systematic review, it is evident that these models should be adapted to incorporate RPV as a control strategy, model multiple cocirculating serotypes, and include livestock population dynamics during the simulations to mimic endemic FMD realistically.

## 4. Discussion

Published original research describing or using SESS model(s) was assessed in this study to identify the specific components of endemic FMD needed to adapt the SESS models for their potential application in FMD endemic settings. It should be emphasized that this current study did not review all models, but only SESS models used for FMD as identified through the database search. Although all the assumptions of these SESS models were reviewed, only the elements necessary for endemicity were considered.

A potential bias could have arisen from restricting this review to the English language, published original research articles, and the specific category of models included, i.e., SESS. Many different types of models could have been selected, ranging from deterministic to automata models to nonspatial models. It should be emphasized that only SESS models were included because of their ability to capture spatiotemporal heterogeneity. We, however, acknowledge the work of all models on FMD, and our decision to include one type of model does not imply that other models were not useful.

Foot-and-mouth disease is endemic in several parts of the world [8–11], and it is associated with substantial economic losses [12–14]. Livestock population dynamics, multiple cocirculating serotypes, and routine prophylactic vaccination (RPV) are critical characteristics of endemic FMD [47, 48, 120, 121]. Published original research describing or using SESS model(s), for understanding the spread of FMD and evaluating control strategies, was qualitatively assessed for their ability to mimic endemic FMD and potential application in endemic settings. It is essential to mention that SESS models identified through the database search have been used in FMD-free settings to understand FMD speed, evaluate effectiveness of different mitigation strategies, assist in developing preparedness plans, and determine economic impacts of FMD. However, none of the identified SESS model is applicable in FMD endemic settings in its current form, taking into consideration the characteristics of endemic FMD [46–49]. The sole reason for this is that these SESS models were developed to mimic the characteristics of FMD in disease-free settings with appropriate set of assumptions that vary from disease-free settings to endemic settings. For instance, stamping-out is often applied as a mitigation strategy in disease-free countries. However, it may not be used as an appropriate mitigation strategy in endemic settings because of limited resources and the inability to pay compensation to livestock owners [121].

All of the SESS models described in this review can model emergency vaccination as a mitigation strategy, but none of them can model RPV which is often cyclically practiced in endemic settings and is relied upon as one of the key measures for control and eradication [120–123]. The reason behind this drawback is very straightforward—models are a simplification of a complex system. Since these SESS models are designed to simplify the system of FMD in disease-free countries, they do not include RPV as a mitigation strategy. The use of the same vaccine in emergency situations could show more effectiveness when compared to RPV in endemic settings [124]. Vaccine factors such as maintenance of cold chain, type of serotype, quality control of vaccine, and duration of immunity greatly influence its effectiveness [46, 125, 126]. Although SESS models include emergency vaccination, the modified SESS model must have an option to model RPV including ability to alter the parameters associated with RPV, such as duration of vaccine immunity, coverage, efficacy, capacity, and frequency of vaccination to more realistically mimic endemic FMD. Adaptation of SESS models to incorporate RPV as an FMD control strategy will extend application of these models to FMD endemic settings. Work along those lines has been initiated by modifying the underlying code of NAADSM to add RPV as a control strategy. NAADSM was chosen for adaptation because of its freely available source code and familiarity of the research team with its simulation architecture [127].

The FMD-free countries usually employ SESS models for preparedness planning and as a decision support tool. To inform these decisions, modelers and epidemiologists do not include multiple cocirculating serotypes; hence, parameters for only one serotype are used to model the spread. Although all the SESS models realistically mimic the underlying system of FMD-free countries, they have a limited ability to be applied to the conditions when the disease is endemic. Multiple cocirculating serotypes, for instance, are common in endemic countries [47, 48, 128], which complicates disease spread and ultimately its control and eradication. The existing SESS model(s) such as NAADSM need to be adapted by changing the underlying code to include options for modeling multiple cocirculating serotypes. Simplifying assumptions should be made to find the balance between model realism vs. complexity while modeling multiple cocirculating serotypes.

In FMD-free countries, when these SESS models are used for preparedness planning, culling is usually employed with or without emergency vaccination. These strict actions in conjunction with disease tracing, surveillance, and availability of resources have led to prompt disease control and subsequent eradication, which results in simulations ending in a very short time and population dynamics having little impact. Therefore, modelers have not considered population dynamics during simulation runs because of it being close to the reality of disease-free settings. However, when SESS models would be used in endemic settings, FMD outbreaks would continue for a longer duration, and it would take longer to control the disease; therefore, eradication cannot be considered a short-term goal. Population turnover is associated with FMD dynamics, such as herd immunity. As newborns are added to the herd, it increases the proportion of unvaccinated naive hosts, thus decreasing herd immunity [120]. Thus, population demographics are also associated with herd susceptibility and infectivity, which are of key significance in disease modeling. Application of SESS models in endemic settings should afford the flexibility to parameterize population dynamics (birth, death processes) to realistically mimic the natural spread of FMD and assess the impact of a changing susceptible population. For example, the underlying code of NAADSM can be modified to add an option to increase the number of animals in the herd when disease simulation runs exceed 365 days and continue this after every 365 days. Some simplifying assumptions should be made, such as applying a country-level growth rate. Subsequently, complexity can be added, such as using a production type-specific growth rate or applying a regional growth rate to account for birth and death process in a specific production type or a geographic region, respectively.

In endemic settings, FMD is associated with substantial economic losses [12–14]. International organizations such as FAO of the UN, OIE, and EuFMD have called for a more targeted control strategy in the “Progressive Control Pathway for FMD” to reduce the disease burden and high economic costs associated with it [16–19]. Endemic countries can benefit from the virtual lab of simulation modeling and evaluate alternative mitigation strategies for FMD control and ultimate eradication. The SESS models, however, should have flexible stop conditions. For instance, a stop condition can be added to NAADSM to “end simulation when prevalence reaches a certain threshold.” Such flexible stop conditions are necessary since the recent demonstration of application of an adapted SESS model in endemic settings have revealed that simulations take quite a long time to end with the in-built “stop condition.” Likewise, flexible “stop conditions” can help endemic countries in evaluating their progress and identify the key actions that can be taken to achieve project-specific goals and milestones.

Model building is a resource-intensive process requiring financial resources as well as technical expertise. Since the process is intensive, it would be wise to adopt a model built for one country to mimic the situation in another country. The model adaptation can be a small change of parameters used in one country to parameters for another country, or it may require changing the underlying code and logic. Before embarking on model adaptation, the researcher should understand the intended purpose of the existing model as well as the adapted model. For instance, NAADSM has been recently adapted for use in FMD endemic settings and its underlying code has been modified to include RPV as a control strategy. The modified modeling framework called Simulation Model For Infectious Animal Diseases in Endemic Regions (SMIAD-ER) has been applied, as a demonstration, in Pakistan to evaluate effective mitigation strategies for FMD control [127]. The reason behind the choice to adapt NAADSM was based on its open source code and past experience of research team members with writing NAADSM and its application.

Utilizing data on the location and population of individual livestock holdings [129] and four scenarios, i.e., baseline, improved movement restrictions, enhanced disease detection, and enhanced RPV, were compared, as a demonstration, to determine the effective strategy for FMD control. The process of model adaptation and application in an endemic setting highlighted the importance of understanding disease epidemiology to incorporate necessary components into the model framework and the necessity of good quality data needed to inform model parameters. Moreover, this demonstration gave confidence in the potential use of SMIAD-ER in endemic settings [127]. The adapted model should, however, undergo rigorous model verification and validation [130].

Model adaptation provides several advantages for both the modeler and the end-user. It provides modelers access to datasets that can be used for model validation. For instance, in our experience, modifying NAADSM to SMIAD-ER has been a driving force for generating and accessing datasets which were otherwise not available such as the data on the location and population of individual livestock holdings and contact networks. The adaptation process also provides a platform to exchange model outcomes among researchers and provides opportunities for end-users such as disease modelers, policymakers, epidemiologists, and experts from endemic countries [131]. Such interactions are essential for modelers to get acquainted with animal production systems to inform the models in a better way [132]. For end-users, it can be relatively cheaper to adapt a model than building one from scratch and gives them technical expertise in epidemiology and disease modeling [131]. The modified model should, however, undergo rigorous verification and validation [130]. The extended use of adapted models will lead to an improvement in FMD control and reduce the global burden of the disease. Finally, model adaptation would be a win-win situation for modelers, epidemiologists, and end-users in endemic settings.

## 5. Conclusions

Simulation modeling is a useful tool to understand the spread and evaluate the mitigation strategies for FMD. Several models have been developed to understand FMD dynamics. The available literature on simulation modeling for FMD is often restricted to FMD-free countries, and existing spatially explicit stochastic simulation models for FMD require modifications before their application in endemic settings. More specifically, these models should be adapted by incorporating components of endemic FMD to mimic endemicity. The adapted models should undergo sensitivity analysis, verification, validation, and agreement analysis for transparency and build credibility. The application of such models in endemic countries can complement FMD control, which will improve livestock health, provide economic gains for producers, and help alleviate poverty and hunger, which will complement efforts to achieve the Sustainable Development Goals.

## Figures and Tables

**Table 1:** SESS models with corresponding citations.

SESS model (number of citations)	Reference of search results
Warwick model (19)	[35, 51–68]
Davis Animal Disease Simulation model (16)	[25, 27, 32, 33, 69–80]
AusSpread model (10)	[28, 40, 41, 81–87]
InterSpread Plus model (9)	[30, 37, 39, 88–93]
North American Animal Disease Spread Model (7)	[29, 36, 94–98]
Australian Animal Disease Spread model (3)	[99–101]
Central Veterinary Institute model (2)	[102, 103]
Traulsen model (2)	[104, 105]
Hayama model (2)	[106, 107]
Multiple models (10)	[78, 86, 108–115]

**Table 2:** Comparison of the features of nine SESS models used for FMD.

SESS model	Multiple serotypes	Emergency vaccination	Routine vaccination	Stamping-out	Population dynamics
Warwick	×	*✓*	×	*✓*	×
DADS	×	*✓*	×	*✓*	×
AusSpread	×	*✓*	×	*✓*	×
ISP	×	*✓*	×	*✓*	×
NAADSM	×	*✓*	×	*✓*	×
AADIS	×	*✓*	×	*✓*	×
CVI	×	*✓*	×	*✓*	×
Traulsen	×	*✓*	×	*✓*	×
Hayama	×	*✓*	×	*✓*	×

## Data Availability

This is a systematic review; hence, no primary data has been used in the manuscript.

## Abbreviations

FMD: Foot-and-mouth disease
SESS: Spatially explicit stochastic simulation (models)
PRISMA: Preferred Reporting Items for Systematic Reviews and Meta-Analyses
DADS: Davis Animal Disease Simulation (model)
DTU-DADS: Denmark Technical University-Davis Animal Disease Simulation (model)
NAADSM: North American Animal Disease Spread Model
IPS: InterSpread Plus (model)
IS: InterSpread (model)
AADIS: Australian Animal Disease Spread (model)
SMIAD-ER: Simulation Model For Infectious Animal Diseases in Endemic Regions
FAO: Food and Agriculture Organization (of the United Nations)
OIE: World Organization for Animal Health
EUFMD: European Commission for the Control of Foot-and-Mouth Disease
PCP-FMD: Progressive Control Pathway for Foot-and-Mouth Disease
GS: Google Scholar
WoS: Web of Science
RPV: Routine prophylactic vaccination
GIS: Geographic information system.

## Supplementary Materials

Supplementary Materials Supplementary material provides PRISMA checklist used in the systematic review process.
