# Supplementary material for: Challenges to the Application of Spatially Explicit Stochastic Simulation Models for Foot-and-Mouth Disease Control in Endemic Settings: A Systematic Review
Source: Comput Math Methods Med. 2020 Nov 21;2020:7841941. doi: 10.1155/2020/7841941 (PMC7700052; doi:10.1155/2020/7841941)
Supplement: Supplementary Materials — Supplementary material provides PRISMA checklist used in the systematic review process. [file 7841941.f1.docx]

**Challenges to the application of spatially explicit stochastic simulation models for foot-and-mouth disease control in endemic settings: A systematic review**

PRISMA checklist

| **Title** | |
| --- | --- |
| Title | The title of the paper identifies it as a systematic review |
| **Abstract** | |
| Abstract | Included |
| **Introduction** | |
| Rationale | Described |
| Objectives | Explicitly stated |
| **Methods** | |
| Protocol and registration | No approved and registered protocol exists  However, it follows PRISMA protocol (PVM publishes systematic review and meta-analysis without approved and registered protocol) |
| Eligibility Criteria | I did specify study characteristics (language, document type, disease, simulation modeling, spatially explicit, stochastic)  Rationale |
| Information sources | List databases, timespan and date searched |
| Search | Described the search strategy |
| Study selection | Screening criteria |
| Data collection process | Use of MS Excel and tabulation |
| Data items | List of variables for which data was sought  Assessment of SESS models and data extraction |
| Risk of bias in individual studies | N/A, not a meta-analysis |
| Summary measures | N/A, not a meta-analysis |
| Synthesis of results | Qualitative synthesis based on assessment of SESS models and then tabulated |
| Risk of bias across studies | N/A, not a meta-analysis |
| Additional analyses | N/A, not a meta-analysis |
| **Results** | |
| Study selection | # identified, # duplicates, # screened, # assessed for eligibility, # excluded (reasons), # included in qualitative synthesis.  Flow diagram  Table 1 |
| Study characteristics | SESS characteristics, Table 2 |
| Risk of bias within studies | N/A |
| Results of individual studies | Table 2 |
| Synthesis of results | N/A, not a meta-analysis |
| Risk of bias across studies | N/A, not a meta-analysis |
| Additional analysis | N/A, not a meta-analysis |
| **Discussion** | |
| Summary of evidence | Identified three key components of missing in the assessed models because of underlying assumptions and reasons behind the design (application in free-settings)  Provided a framework for model adaptation |
| Limitations | Described that only assessed SESS models used for FMD  Could be more models (not SESS, not published original research, not used for FMD) |
| Conclusions | Described |
| **Funding** | |
| Funding | No specific funding |
